# Clinical Characteristics and Determinants of Complications in Pediatric Herpes Zoster Requiring Hospitalization

**DOI:** 10.3390/children13050640

**Published:** 2026-05-03

**Authors:** Anuța Bilașco, Anca Cristina Drăgănescu, Victor Daniel Miron, Cristina Ramona Rădulescu, Diana Maria Băncilă, Ana-Maria Tudor, Angelica Constanța Vișan, Doina-Anca Pleșca

**Affiliations:** 1 Carol Davila University of Medicine and Pharmacy, 050474 Bucharest, Romaniaana.tudor@umfcd.ro (A.-M.T.); doina.plesca@umfcd.ro (D.-A.P.); 2 National Institute for Infectious Diseases “Prof. Dr. Matei Balș”, 021105 Bucharest, Romania

**Keywords:** children, herpes zoster, varicella-zoster virus, complications, hospitalization, zoster sine herpete

## Abstract

Background: Herpes zoster (HZ) is uncommon in children and is typically considered a mild disease. However, hospitalized cases may be associated with complications. Data on herpes zoster in children in Eastern Europe are limited. This study aimed to describe the clinical features, complications, and factors associated with severity in hospitalized pediatric HZ. Methods: We conducted a retrospective cohort study including children (<18 years) hospitalized with HZ between January 2015 and December 2025, at a tertiary infectious diseases center in Bucharest, Romania. Demographic, clinical, laboratory, and outcome data were extracted from medical records. Univariable and multivariable analyses were performed to identify factors associated with complications and prolonged hospital stay. Results: Among 612 pediatric HZ cases, 92 (15.0%) required hospitalization. The median age was 8.8 years (IQR 4.8–13.2), and 52.2% were male. Overall, 43.5% developed complications, most commonly bacterial superinfection (30.4%), followed by neurological and ophthalmological involvement. Herpes zoster sine herpete was identified in 4.3% of cases and was associated with central nervous system involvement. Headache (OR = 4.34, *p* = 0.025) and lymphopenia (OR = 3.01, *p* = 0.020) were independently associated with complications. Patients with complications had longer hospital stays (median of 6 vs. 4 days, *p* = 0.002). In multivariable analysis, complications, immunocompromised status, and chronic conditions were associated with prolonged hospitalization. Conclusions: Herpes zoster in children is generally mild and has a favorable prognosis; however, hospitalized cases are often associated with complications, especially bacterial superinfections and neurological involvement. These findings derive from a selected population and highlight the role of clinical and host-related factors in shaping outcomes in hospitalized pediatric herpes zoster.

## 1. Introduction

Varicella-zoster virus (VZV), also known as human herpesvirus type 3, is an enveloped double-stranded DNA virus belonging to the *Herpesviridae* family, subfamily *Alphaherpesvirinae* [1]. Primary infection typically occurs during childhood and manifests as varicella (chickenpox), after which the virus establishes lifelong latency in the dorsal root, cranial nerves or autonomic ganglia. Reactivation of latent VZV leads to herpes zoster (HZ), a condition associated with declining or impaired cell-mediated immunity [2,3]. HZ develops in approximately 30% of individuals with prior VZV infection, with a global incidence estimated at 3–5 cases per 1000 person-years [4,5].

Although the disease predominantly affects older adults, it can also occur in children, particularly in the presence of immunosuppression or other predisposing conditions. In the pediatric population, the incidence is estimated at approximately 0.45 cases per 1000 children under 14 years of age, remaining substantially lower than that observed in adults [5,6]. In immunosuppressed children, the risk of VZV reactivation is 5–6 times higher [5]. Identified pediatric risk factors include cellular immunosuppression, early primary infection, intrauterine exposure to VZV, chronic underlying disease, malnutrition, and asthma [5,7,8].

VZV reactivation can occur in both immunocompetent and immunosuppressed children. Immunodeficiency screening is not routinely required in all hospitalized herpes zoster patients. Clinical signals suggesting underlying immunodeficiency include recurrent or disseminated HZ, lesion progression or persistence despite antiviral therapy, new lesions appearing >1 week after onset, frequent infections from other causes, and positive family history of primary immunodeficiency [9].

Clinically, HZ is characterized by a unilateral vesicular rash distributed along one or more dermatomes, frequently accompanied by neuropathic pain. In rare cases, VZV reactivation may occur without cutaneous involvement, affecting the central nervous system or the gastrointestinal tract. This clinical entity is referred to as herpes zoster sine herpete [8,10]. Although the diagnosis is usually clinical, atypical presentations may require confirmation by real-time polymerase chain reaction (RT-PCR) detection of VZV DNA from vesicular fluid or cerebrospinal fluid (CSF) [11,12]. While most pediatric cases follow a benign course, complications may occur, including bacterial skin infections, neurological involvement (such as meningitis or encephalitis), and ophthalmologic manifestations [5,8,10,13].

Despite the widespread use of varicella vaccination in many countries, VZV infection continues to represent a relevant public health concern. In Romania, where varicella vaccination is not included in the National Immunization Program, vaccination coverage is low and is not systematically monitored. Varicella incidence therefore remains substantial, reflecting ongoing viral circulation in the pediatric population. After a marked decline during the COVID-19 pandemic (2020–2021), likely related to reduced social contact, incidence increased again in the post-pandemic period, reaching 162 cases per 100,000 population in 2024, although remaining slightly below pre-pandemic levels as reported in 2019 (171.4 per 100,000 population) [14]. In contrast, HZ is not systematically reported at the national level, and epidemiological data regarding VZV reactivation in the pediatric population remain limited. At the same time, there is a lack of well-defined, evidence-based, pediatric-specific treatment guidelines. In clinical practice, therapeutic decisions are often extrapolated from adult recommendations, largely due to the low incidence of the disease in children and the limited availability of pediatric-specific evidence.

In this context, the present study aimed to characterize the clinical features, spectrum of complications, and outcomes of pediatric herpes zoster requiring hospitalization in a tertiary referral center from Romania. In addition, we evaluated factors associated with prolonged hospitalization, with particular attention towards clinical and laboratory markers that may reflect disease severity.

## 2. Methods

### 2.1. Study Design and Participants

We conducted a retrospective, observational study including pediatric patients aged under 18 years who were hospitalized in the National Institute for Infectious Diseases “Prof. Dr. Matei Balș” (NIID) from Bucharest, Romania, between 2015 and 2025, with a diagnosis of HZ. NIID is the main infectious diseases hospital in Romania, a national reference center for the management of infectious diseases, caring for both adults and children, with approximately 740 inpatient beds (including 309 pediatric beds) and managing an average of 18,000 pediatric hospitalizations annually. During the COVID-19 pandemic, the institute functioned primarily as a dedicated COVID-19 facility, with a substantial reduction in pediatric admissions, which may have influenced the observed distribution of cases over time.

Medical records were reviewed to extract demographic, clinical, laboratory, complications and treatment data. Eligible cases were identified by querying the hospital’s electronic registry for all pediatric hospitalizations (age under 18 years) during the study period (1 January 2015–31 December 2025), using ICD-10 diagnostic codes (B02.0-B02.8) and clinical terms corresponding to VZV reactivation. For each potentially eligible record, the complete medical chart was independently reviewed by two investigators using a standardized data extraction form. Cases were included if they met the operational definition of VZV reactivation (HZ, defined as a clinically documented dermatomal vesicular rash or zoster sine herpete) and if hospitalization was primarily due to the acute episode. Hospitalization decisions were based on clinical severity, presence of complications, comorbidities, or the need for intravenous antiviral therapy according to institutional standards. Discrepancies between investigators were resolved by consensus.

Demographic data, comorbidities and immune status, clinical characteristics of the episode, laboratory findings, administered treatment and outcomes (including complications and length of stay) were collected. Readmissions for the same episode were aggregated and considered a single episode. For each variable, the proportion of missing data was assessed, and analyses were performed on available data, explicitly reporting the number of observations included in each analysis. Patients were grouped by presence or absence of complications for comparative analyses.

### 2.2. Definitions

The diagnosis of HZ was established clinically, based on the presence of a characteristic dermatomal vesicular rash, often accompanied by pain and itching. Polymerase chain reaction (PCR) testing for varicella-zoster virus DNA was performed in selected cases with atypical presentations, including suspected central nervous system involvement or herpes zoster sine herpete.

Dermatomal distribution of HZ was categorized as cervical, thoracic, or lumbosacral based on the initial site of eruption. Cranial nerve involvement was recorded separately and included ophthalmic (V1), maxillary/mandibular (V2–V3), combined trigeminal involvement, and facial nerve involvement (Ramsay Hunt syndrome). Multidermatomal disease was defined as the involvement of more than two dermatomes.

A diagnosis of herpes zoster sine herpete was suspected in patients presenting with compatible clinical features, particularly neurological involvement suggestive of varicella-zoster virus reactivation and confirmed by the detection of VZV DNA in cerebrospinal fluid. Initial testing used a multiplex RT-PCR assay (BioFire^®^ FilmArray^®^, BioFire™ Diagnostics, Inc., Salt Lake City, UT, USA), followed by quantitative real-time RT-PCR for VZV DNA in the same CSF samples. In our cohort, all cases that presented with central nervous system involvement underwent virological confirmation by RT-PCR.

Lymphopenia was defined according to age-adjusted pediatric reference ranges used by the local laboratory (<3000 cells/mm^3^ for 0–2 years; <2000 cells/mm^3^ for 2–6 years; <1500 cells/mm^3^ for 6–18 years).

Serological testing was performed as part of routine clinical evaluation to assess prior exposure and immune status. Specific anti-VZV IgM and IgG antibodies were measured using an enzyme-linked immunosorbent assay (ELISA), according to the manufacturer’s instructions. Given the limited sensitivity and specificity of serological markers in VZV reactivation, the results were interpreted in the clinical context and were not used to establish the diagnosis.

Complications were recorded if documented in the medical record, and multiple complications could occur in the same patient. These included bacterial skin superinfection, ophthalmological involvement (conjunctivitis, keratitis, keratoconjunctivitis), Ramsay Hunt syndrome, meningitis (clinical syndrome with CSF pleocytosis, with PCR confirmation when available), peripheral facial palsy unrelated to Ramsay Hunt syndrome, and other complications such as acute hepatitis, radiculomyelitis, or hyperalgesia. Bacterial superinfection was defined primarily based on clinical criteria, with microbiological confirmation available only in a subset of cases, reflecting standard clinical practice. All patients with suspected ophthalmological involvement underwent specific evaluation, and diagnoses were confirmed by an experienced ophthalmologist.

Routine screening for primary immunodeficiency is not recommended in all patients with HZ and is typically guided by clinical features suggestive of underlying immune dysfunction. As such, systematic screening for primary immunodeficiency was not performed in all patients included in this study. Immunocompromised status was therefore defined based on documented underlying conditions and/or ongoing immunosuppressive treatment recorded in the medical charts. All patients underwent HIV testing as part of routine clinical evaluation.

### 2.3. Statistical Analysis

Statistical analysis was performed using Jamovi (version 2.6.44, open-source) and Microsoft Excel (Microsoft Office 365, Microsoft Corporation, Redmond, WA, USA). Categorical variables were summarized as counts and percentages, while continuous variables were assessed for normality using the Shapiro–Wilk test. Continuous variables with non-normal distributions were described using medians and interquartile ranges (IQR), whereas normally distributed variables were summarized as means and standard deviations, as appropriate. Comparisons between groups were performed using the chi-square or Fisher’s exact test for categorical variables and Student’s *t*-test or Mann–Whitney U test for continuous variables, as appropriate. Odds ratios (ORs) with 95% confidence intervals (CIs) were calculated for selected bivariate associations. A multivariable logistic regression model was constructed to identify factors independently associated with the occurrence of complications, with results reported as ORs and 95% CIs. Variables included in the multivariable model were selected based on clinical relevance, univariable association, and model parsimony. Cranial nerve involvement was not included together with headache due to potential overlap and collinearity. Univariable analyses were performed to assess the association between clinical and laboratory variables and length of hospital stay (LOS). Variables identified in univariable analysis (*p* < 0.10), along with clinically relevant factors, were included in a multivariable linear regression model. Due to the right-skewed distribution of LOS, a logarithmic transformation was applied prior to analysis. Regression coefficients were back-transformed and expressed as percentage change in LOS. Additionally, a sensitivity analysis was performed excluding patients with herpes zoster sine herpete, as these cases represent atypical presentations and were all associated with central nervous system complications in our cohort. Missing data were minimal and instances were handled using complete-case analysis. A *p*-value < 0.05 was considered statistically significant.

### 2.4. Ethics

This study was conducted in accordance with institutional and national ethical standards and in compliance with the Declaration of Helsinki. Given the retrospective design and the use of routinely collected clinical data, the requirement for informed consent was waived in accordance with local regulations and institutional policy. The study protocol was approved by the Ethics Committee of the National Institute for Infectious Diseases “Prof. Dr. Matei Bals” (approval number C06634/20.06.2025).

## 3. Results

### 3.1. Study Population and Baseline Characteristics

A total of 612 children were diagnosed with HZ during the study period, and only 92 (15.0%) of them required hospitalization. Only hospitalized cases were included in the analysis. Median age was 8.8 years (IQR: 4.8–13.2 years) with a slight predominance of males (52.2%, *n* = 48, Table 1). Nearly two-thirds of cases (60.9%, *n* = 56) occurred during the pre-pandemic period. In total, 29 children (31.5%) had at least one chronic underlying condition, and 20.7% (*n* = 19) were classified as immunocompromised, most commonly due to corticosteroid therapy (10.9%, n = 10). Regarding VZV exposure history, a previous known episode of varicella was reported in most patients (79.3%, *n* = 73), while intrauterine exposure to VZV was reported in 8.7% (*n* = 8). Only one patient had a documented history of vaccination (Table 1).

### 3.2. Clinical Presentation, Topographic Distribution, Laboratory Findings and Antiviral Treatment

**Figure 1:**
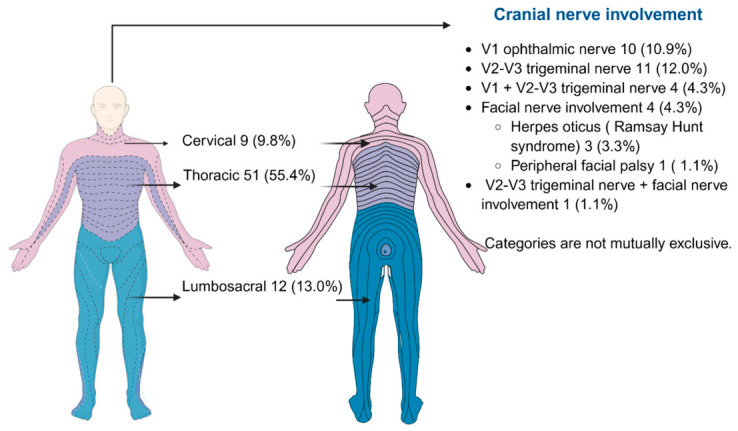
Topographic distribution of pediatric herpes zoster cases. The figure depicts cutaneous involvement across cervical, thoracic and lumbosacral regions and cranial nerve involvement. V1, ophthalmic nerve; V2–V3, maxillary and mandibular nerves. Percentages are calculated using the total cohort (N = 92); categories are not mutually exclusive. Figure created by BioRender.com.

Dermatomal pain (*n* = 34, 37.0%), fever (*n* = 29, 31.5%), and headache (*n* = 19, 20.7%) were the most frequent clinical symptoms at admission (Table 2). Thoracic dermatomes were mostly involved (*n* = 51, 55.4%, Figure 1). Overall, non-cranial HZ was predominant (*n* = 72, 78.3%), while cranial involvement was observed in 20 patients (21.7%), and herpes zoster sine herpete was identified in 4 cases (4.3%).

Lymphopenia was the most frequent laboratory abnormality (39.1%), whereas other hematological changes, including leukocytosis (17.4%) and anemia (16.3%), were less commonly observed. VZV serological testing was available for 57 of the 92 patients. IgG antibodies were identified in 47/57 cases (82.5), while IgM antibodies were positive in 19/57 (33.3%). Among the 10 patients lacking detectable IgG, two had documented intrauterine exposure to VZV, four were immunocompromised children, and one had prior varicella. For the remaining 3 patients, detailed information regarding previous exposure was not available in the medical records; however, all these cases had a clinical presentation highly suggestive of herpes zoster.

During hospitalization, antiviral therapy was administered according to the clinical presentation; 27 patients (29.3%) received intravenous acyclovir, 61 (66.3%) received oral treatment, and 4 patients (4.3%) did not receive antiviral therapy. The median length of hospital stay for the entire cohort was 5 days (IQR: 3, 7 days) (Table 2).

### 3.3. Complications and Associated Risk Factors

Overall, 40 patients (43.5%) had at least one complication during hospitalization (Table 3). Bacterial superinfection was the most common complication, followed by ophthalmological and neurological manifestations. Zoster sine herpete was identified in 4 cases (4.3%): three had acute meningitis confirmed by VZV DNA detection in CSF and one had thoracic radiculomyelitis.

In univariable analysis (Table 4), headache and lymphopenia were significantly more frequent among patients with complications compared to those without (35.0% vs. 9.6%, OR = 5.06, 95%CI: 1.64–15.6, *p* = 0.004; and 55.0% vs. 26.9%, OR = 3.32, 95%CI: 1.38–7.95, *p* = 0.006, respectively). After excluding herpes zoster sine herpete cases, headache remained significantly associated with complications (OR = 3.62, 95% CI: 1.12–11.71, *p* = 0.032) and was more frequent in patients with cranial nerve involvement compared to those with non-cranial disease (37.5% vs. 12.5%, OR = 4.21, 95%CI: 1.251–4.1, *p* = 0.016).

Fever and multidermatomal involvement were also observed more commonly in this group, although these associations did not reach statistical significance (42.5% vs. 23.1%, *p* = 0.070; and 40.0% vs. 21.2%, *p* = 0.065, respectively). Intravenous acyclovir was more frequently administered in patients with complications compared to those without (42.5% vs. 19.2%, *p* = 0.021). In addition, patients who developed complications had a significantly longer hospital stay compared to those without (median of 6 vs. 4 days, *p* = 0.002), while age distribution did not differ between groups.

In multivariable logistic regression analysis (Table 5), headache (OR = 4.34, 95% CI: 1.201–5.66, *p* = 0.025) and lymphopenia (OR = 3.01, 95% CI: 1.19–7.60, *p* = 0.020) remained independently associated with the occurrence of complications.

Given the observed difference in LOS, we further investigated factors associated with prolonged hospitalization in univariable analysis (Table 6). Immunocompromised patients and those with underlying chronic conditions experienced longer hospitalization durations (7 [6–8] vs. 5 [3–6] days, *p* < 0.001; and 6.5 [6–9] vs. 4 [3–6] days, *p* < 0.001, respectively). Lymphopenia was also associated with an increased length of stay in univariable analysis (6 [4.75–9.25] vs. 4 [3–6] days, *p* = 0.005).

Variables identified in univariable analysis, along with clinically relevant factors, were subsequently included in the multivariable linear regression model (Table 7). In this model, complications remained the strongest predictor of prolonged hospitalization, being associated with a 49.5% increase in length of stay (*p* < 0.001). Immunocompromised status, fever at admission, and chronic conditions were also independently associated with longer hospital stay (+39.2%, *p* = 0.027; +31.4%, *p* = 0.024; and +34.4%, *p* = 0.024, respectively). Although lymphopenia was associated with longer hospital stay in univariable analysis, this association was not retained after adjustment (*p* = 0.127), suggesting potential confounding by other clinical factors.

## 4. Discussion

Although herpes zoster is relatively uncommon in children and most cases are mild and managed in outpatient settings, our findings highlight a different clinical profile among hospitalized patients. In this retrospective cohort, we identified a substantial burden of complications, affecting nearly half of the cases (43.5%). These findings contribute to the limited body of evidence on pediatric HZ requiring hospitalization and highlight specific clinical and laboratory markers associated with outcomes.

This cohort represents a selected population as only hospitalized cases were included, limiting generalizability to the broader pediatric population. The proportion of hospitalized cases (15% of all pediatric HZ presentations evaluated in our center) is consistent with previous reports indicating that most pediatric HZ cases are managed in outpatient settings, with hospitalization reserved for complicated or atypical presentations [15,16,17,18].

The median age of 8.8 years and the predominance of cases in the 5–9-year age group align with known epidemiological data, suggesting that pediatric HZ most commonly occurs in school-aged children. The incidence of HZ in children increases with age, being lowest in preschool children and increasing through school years and into adolescence. Population-based studies from both the pre- and post-varicella vaccination eras consistently show that most pediatric herpes zoster cases occur in children aged 5–12 years and adolescents, with the highest rates observed in the 10–19-year age group [13,19,20,21]. In the post-vaccination era, there is evidence of a shift toward younger ages at presentation among vaccinated children, but the mean age of onset in most cohorts remains around 8–10 years, and school-aged children continue to represent a substantial proportion of cases [15,21,22]. While HZ can occur at any age in childhood, the risk is highest in those who had varicella at a very young age or are immunocompromised. The overall burden of disease is now lower in vaccinated populations, but school-aged children remain a commonly affected group [23,24].

The distribution of VZV exposure observed in our cohort suggests a predominance of natural infection, which can be explained by the low vaccination coverage in Romania where varicella vaccination is not included in the National Immunization Program. Consequently, the very small percentage of vaccinated patients did not allow an analysis of vaccine strain VZV reactivation. In contrast, in countries with routine varicella vaccination, the epidemiology of pediatric herpes zoster is different, likely due to reduced circulation of wild-type VZV and changes in immune-boosting following vaccination [13,19,20].

The predominance of thoracic dermatomal involvement (55.4%) and non-cranial distribution (78.3%) in our study is consistent with classical descriptions of HZ across age groups. Cranial nerve involvement, although less frequent (21.7%), remains clinically significant due to its association with increased morbidity, particularly ophthalmic involvement and Ramsay Hunt syndrome [25]. Notably, only 37% of patients reported dermatomal pain, which is lower than typically described in adults. Acute neuropathic pain and postherpetic neuralgia are significantly less common and less severe in pediatric HZ than in adults. Most children experience pruritus or mild discomfort rather than persistent or severe pain, and the majority of cases resolve without complications or chronic pain [18,21,26]. Postherpetic neuralgia is rare in immunocompetent and vaccinated children, and when it does occur, it is typically mild and short-lived, with studies consistently showing very low rates of pain lasting beyond one month [21,26,27]. The relative infrequency and mildness of neuropathic pain in pediatric HZ may contribute to under-recognition or diagnostic delays, especially in atypical or painless presentations. Neuropathic pain, although typically less severe than in adults, can manifest as irritability, agitation, and sleep disturbances, negatively impacting quality of life in children [28,29]. Clinicians may not suspect herpes zoster in the absence of significant pain, and atypical cases, such as those presenting with pruritus, mild discomfort, or rash without pain, can be overlooked or misdiagnosed [18,30,31]. This is particularly relevant in the post-vaccination era, where the clinical course is milder and pain is even less common among vaccinated children [18,21]. Recognition of this age-related difference in symptoms is important for timely diagnosis and management, especially in cases with cranial nerve involvement or atypical dermatomal patterns, where morbidity may be higher despite the absence of classic neuropathic pain.

Serology was available only in a subset of patients and showed detectable IgG in most tested children. A minority with negative IgG included cases with documented intrauterine exposure or prior varicella, as well as immunocompromised children, indicating that serology may not reliably exclude prior exposure in selected clinical contexts; clinical assessment and molecular testing remain important, particularly in atypical cases [32,33].

Neurological complications, including meningitis, radiculomyelitis, and peripheral facial palsy, show the neurotropic nature of VZV. Importantly, we identified four cases (4.3%) of herpes zoster sine herpete, three presenting as VZV meningitis and one as radiculomyelitis. This entity, characterized by VZV reactivation in the absence of cutaneous lesions, remains underdiagnosed, particularly in children, and requires a high index of suspicion and virological confirmation (PCR from CSF) [34]. The recognition of zoster sine herpete expands the clinical spectrum of pediatric HZ, and VZV reactivation should be considered in cases of aseptic meningitis or unexplained neurological syndromes, even in the absence of a rash [35]. Herpes zoster sine herpete may also present with visceral involvement, including rare cases of abdominal forms caused by VZV reactivation in autonomic or mesenteric ganglia. These presentations can manifest as prolonged, unexplained abdominal pain in the absence of cutaneous lesions and may mimic common gastrointestinal conditions [36,37,38]. In our cohort, no case of visceral herpes zoster was observed, even in immunosuppressed children.

Headache and lymphopenia emerged as clinically relevant factors associated with complications. Headache, in particular, showed a robust association with complications and was especially prevalent in cranial HZ and in all zoster sine herpete cases, most of which had confirmed CNS involvement. This supports the hypothesis that headache may represent an early clinical marker of neurological involvement rather than a nonspecific symptom. This association remained significant even after excluding cases of herpes zoster sine herpete, indicating that it is not solely driven by atypical presentations. Previous reports have described VZV meningitis, encephalitis, and myelitis occurring without skin lesions, particularly in cases involving cranial nerve ganglia or presenting as herpes zoster sine herpete. Both pediatric and adult case series have emphasized that headache may be an early or predominant manifestation of VZV-related central nervous system disease, supporting its role as a clinical warning sign [10,39,40,41]. Although pediatric data directly linking lymphocyte counts to complicated HZ are limited, evidence from immunologically vulnerable populations supports this association [7,42]. In adult patients with systemic lupus erythematosus, lymphopenia has been reported as an independent risk factor for complicated herpes zoster. In this context, the association observed in our cohort between lymphopenia and complicated disease appears biologically plausible. Therefore, lymphopenia could represent a simple and accessible marker to aid risk stratification in pediatric viral infections [42,43,44,45].

The use of IV acyclovir among patients with complications likely reflects greater baseline disease severity rather than a causal relationship, as intravenous therapy is typically reserved for patients with severe or complicated presentations. These findings are consistent with previous reports where the use of IV antiviral treatment is primarily driven by disease severity and clinical indication, rather than being independently associated with outcomes [5]. Due to the retrospective design, detailed data regarding the duration of intravenous versus oral antiviral therapy were not consistently available, particularly for treatment administered prior to admission.

In addition to the analysis of complications, we further explored factors associated with prolonged hospitalization as an indicator of disease burden and healthcare resource utilization. Complications emerged as the strongest determinant of length of stay, which was to be expected and likely reflects overall disease severity and the need for more intensive management. Immunocompromised status and the presence of chronic conditions were also independently associated with longer hospitalizations. Notably, the distribution of hospital stay in these patients was consistently shifted toward higher values, suggesting a uniform impact rather than being driven solely by extreme cases. These findings are consistent with previous reports, where disease severity, complications, and underlying comorbidities have been identified as major determinants of a prolonged hospital stay [10,17,46].

Fever at admission was not significantly associated with length of stay in univariable analysis but became significant in the multivariable model. This suggests that fever may act as a marker of disease severity whose effect becomes apparent after adjustment for other clinical factors. The wider interquartile range observed in febrile patients further supports the presence of greater variability and potentially more severe cases within this group.

Lymphopenia was associated with longer hospitalization in univariable analysis but did not remain an independent predictor after adjustment. This finding suggests that lymphopenia may reflect an underlying inflammatory or immunological response associated with more severe disease rather than acting as a direct determinant of prolonged hospitalization. Its association with length of stay is therefore likely mediated through its relationship with complications and overall disease severity.

Our findings reinforce that pediatric HZ, although less frequent than in adults, can sometimes be associated with significant morbidity, particularly in hospitalized cases. They also highlight the importance of varicella vaccination in preventing primary infection. In the context of declining vaccination coverage in Europe and the re-emergence of vaccine-preventable diseases such as measles, sustained immunization efforts remain essential [47]. In Romania, where varicella vaccination is not included in the national immunization program, the burden of VZV-related disease remains substantial.

This study has several limitations. First, its retrospective design relies on the accuracy and completeness of medical records, which may have led to underreporting of certain clinical features, particularly subjective symptoms such as pain or mild complications. Second, the study population includes only hospitalized patients from a single tertiary referral center, introducing a potential selection bias toward more severe cases and limiting the generalizability of our findings to the broader pediatric population. In addition, the lack of clinical, biological, and outcome data for patients managed in outpatient settings precludes a comprehensive comparison across the full spectrum of disease severity and may further increase selection bias. Third, virological confirmation was not systematically performed in all cases, particularly in typical cutaneous presentations, which may have led to misclassification or underdiagnosis of atypical forms such as zoster sine herpete. Fourth, the relatively small sample size may have reduced the statistical power to detect associations for less frequent risk factors and limited the robustness of multivariable analyses. Additionally, certain potentially relevant variables, such as detailed immunological status, prior antiviral treatment, data regarding the total duration of antiviral therapy or precise timing of symptom onset, were not consistently available, which may have introduced residual confounding. Finally, long-term outcomes, including postherpetic neuralgia or persistent neurological deficits, were not systematically assessed, precluding evaluation of the full burden of the disease.

## 5. Conclusions

In conclusion, pediatric herpes zoster requiring hospitalization represents a selected subgroup of patients, in which complications, including neurological involvement and atypical presentations such as zoster sine herpete, may be observed. However, herpes zoster in children is generally mild, with a favorable prognosis and very low mortality, and most cases are managed in outpatient settings. In this cohort, headache and lymphopenia were associated with the occurrence of complications and may aid in early risk stratification. These findings should be interpreted with caution, as they may reflect underlying disease processes or the acute phase of infection rather than independent predictors of severity. Prolonged hospitalization was mainly driven by disease severity, particularly complications and underlying conditions.

Increased awareness of the diverse clinical spectrum of VZV reactivation in children, combined with improved surveillance and vaccination strategies, is essential to reduce disease burden. Further multicenter and prospective studies are needed to better define epidemiology, risk stratification, and management of pediatric herpes zoster.

## Figures and Tables

**Table 1:** General characteristics of patients.

Characteristics	N (%)
Biological sex	
Male	48 (52.2)
Female	44 (47.8)
Period	
Pre-pandemic (2015–2019)	56 (60.9)
Pandemic/post-pandemic (2020–2025)	36 (39.1)
Age groups	
<1 year	2 (2.2)
1–4 years	21 (22.8)
5–9 years	31 (33.7)
10–14 years	24 (26.1)
15–17 years	14 (15.2)
Underlying conditions	
At least one chronic condition	29 (31.5)
Hematologic/oncologic disease	5 (5.4)
Immune diseases	7 (7.6)
HIV infection	5 (5.4)
Metabolic disorders	13 (14.1)
No chronic condition	63 (68.5)
Immunocompromised status	
Immunocompromised	19 (20.7)
Chemotherapy	5 (5.4)
Biologic therapy	1 (1.1)
Corticosteroid therapy	10 (10.9)
VZV exposure history	
Intrauterine exposure	8 (8.7)
History of varicella	73 (79.3)
VZV vaccination	1 (1.1)

Data are presented as number (percentage). Percentages are calculated using the total study population (N = 92). HIV, human immunodeficiency virus, VZV, varicella-zoster virus.

**Table 2:** Clinical presentation, topographic distribution, laboratory findings, and antiviral treatment in hospitalized pediatric herpes zoster.

Characteristics	N (%)
Clinical features	
Dermatomal pain	34 (37.0)
Fever	29 (31.5)
Headache	19 (20.7)
Pruritus	16 (17.4)
Sleepiness	5 (5.4)
Restlessness	4 (4.3)
Topography of herpes zoster	
Non-cranial herpes zoster	72 (78.3)
Cranial herpes zoster	20 (21.7)
Multidermatomal involvement	27 (29.3)
Herpes zoster sine herpete	4 (4.3)
Cranial nerve involvement (n = 20)	
Zoster ophthalmicus (V1)	10 (50.0)
Trigeminal nerve (V2–V3)	13 (65.0)
Combined V1 + V2–V3	4 (20.0)
V2–V3 + facial nerve	1 (5.0)
Facial nerve	4 (20.0)
Laboratory findings	
Leukocytosis	16 (17.4)
Leukopenia	5 (5.4)
Lymphopenia	36 (39.1)
Neutrophilia	4 (4.3)
Anemia	15 (16.3)
Serology (available cases)	
IgG antibodies for VZV (n = 57)	47 (82.5)
IgM antibodies for VZV (n = 57)	19 (33.3)
Antiviral treatment	
Oral acyclovir	61 (66.3)
Intravenous acyclovir	27 (29.3)
No antiviral treatment	4 (4.3)

Data are presented as number (percentage). Percentages are calculated using the total study population (N = 92), except for cranial nerve involvement, where values are expressed relative to the number of affected cases (N = 20). IgG, immunoglobulin G; IgM, immunoglobulin M; VZV, varicella-zoster virus; V1, ophthalmic branch of the trigeminal nerve; V2–V3, maxillary and mandibular branches of the trigeminal nerve.

**Table 3:** Types of complications in hospitalized pediatric herpes zoster patients.

Type of Complication	N (%)
Any complication	40 (43.5)
≥2 complications	4 (4.3)
Bacterial superinfection	28 (30.4)
Ophthalmological involvement	9 (9.8)
Meningitis	4 (4.3)
Ramsay Hunt syndrome	3 (3.3)
Peripheral facial palsy (non-Ramsay)	1 (1.1)
Hyperalgesia	2 (2.2)
Radiculomyelitis	1 (1.1)
Acute hepatitis	2 (2.2)

Data are presented as number (percentage). Percentages are calculated using the total study population (N = 92). Patients may have presented with more than one complication.

**Table 4:** Clinical and laboratory characteristics according to complications.

Variable	Complications, N = 40 n (%)	No Complications, N = 52 n (%)	OR (95% CI)	*p*-Value
Age (years), median (IQR)	6.6 (4.2–13.3)	9.2 (5.3–12.3)	NA	0.714
Length of stay (days), median (IQR)	6 (5–9)	4 (3–6)	NA	0.002
Male sex	25 (62.5)	23 (44.2)	2.10 (0.90–4.88)	0.082
Chronic conditions	13 (32.5)	16 (30.8)	1.08 (0.45–2.63)	1.000
Immunocompromised status	8 (20.0)	11 (21.1)	0.93 (0.34–2.59)	0.892
Dermatomal pain	20 (50.0)	14 (26.9)	2.71 (1.14–6.49)	0.023
Fever	17 (42.5)	12 (23.1)	2.46 (1.00–6.06)	0.070
Headache	14 (35.0)	5 (9.6)	5.06 (1.64–15.6)	0.004
Pruritus	9 (22.5)	7 (13.4)	1.87 (0.63–5.54)	0.250
Sleepiness	2 (5.0)	3 (5.7)	2.03 (0.32–12.8)	0.649
Restlessness	1 (2.5)	3 (5.7)	0.42 (0.04–4.19)	0.630
Non-cranial herpes zoster	23 (57.5)	45 (86.5)	0.21 (0.07–0.58)	0.020
Cranial herpes zoster	13 (32.1)	7 (17.5)	3.10 (1.10–8.72)	0.028
Multidermatomal involvement	16 (40.0)	11 (21.2)	2.48 (0.99–6.22)	0.065
Leukocytosis	5 (12.5)	11 (21.2)	0.53 (0.17–1.68)	0.278
Leukopenia	1 (2.5)	4 (7.6)	0.31 (0.03–2.87)	0.380
Lymphopenia	22 (55.0)	14 (26.9)	3.32 (1.38–7.95)	0.006
Neutrophilia	2 (5.0)	4 (7.6)	1.58 (0.27–9.11)	0.604
Anemia	7 (17.5)	8 (15.4)	1.17 (0.38–3.54)	0.784

Data are presented as number (percentage) or median (interquartile range, IQR). *p*-values were obtained using chi-square or Fisher’s exact test for categorical variables and Mann–Whitney U test for continuous variables. OR, odds ratio; CI, confidence interval; NA, not applicable.

**Table 5:** Multivariable logistic regression for predictors of complications.

Variable	OR (95% CI)	*p*-Value
Fever	1.16 (0.39–3.45)	0.784
Headache	4.34 (1.20–15.66)	0.025
Lymphopenia	3.01 (1.19–7.60)	0.020
Immunocompromised status	0.95 (0.31–2.86)	0.923
Intercept	0.35 (0.18–0.71)	0.003

OR, odds ratio; CI, confidence interval. Outcome: presence of complications.

**Table 6:** Univariable analysis of factors associated with length of hospital stay.

Variable	LOS, Median (IQR), Days	*p*-Value
Complications	6 (5–9) vs. 4 (3–6)	0.002
Immunocompromised status	7 (6–8) vs. 5 (3–6)	<0.001
Chronic conditions	6.5 (6–9) vs. 4 (3–6)	<0.001
Fever at admission	6 (3–12) vs. 5 (3–6)	0.054
Lymphopenia	6 (4.75–9.25) vs. 4 (3–6)	0.005
≥2 dermatomes involved	6 (4–7.5) vs. 5 (3–7)	0.482

Data are presented as median (interquartile range, IQR). *p*-values were calculated using the Mann–Whitney U test.

**Table 7:** Log-transformed linear regression: factors associated with length of hospital stay.

Predictor	β (log)	SE	95% CI (log)	% Change in LOS	*p*-Value
Complications	0.402	0.111	0.184–0.620	49.5% (20.2–85.9)	<0.001
Immunocompromised status	0.331	0.147	0.043–0.619	39.2% (4.4–85.7)	0.027
Fever	0.273	0.119	0.040–0.506	31.4% (4.0–65.9)	0.024
Chronic conditions	0.296	0.129	0.044–0.548	34.4% (4.5–73.0)	0.024
Lymphopenia	0.175	0.114	−0.048–0.398	19.1% (−4.7–48.9)	0.127

Dependent variable: log (length of stay). Percent change computed as (e^β−1^) × 100%. CI, confidence interval; SE, standard error; LOS, length of stay. Model R^2^ = 0.365 (adjusted R^2^ reported in text).

## Data Availability

The datasets supporting this research are available upon request from the corresponding author.

## Abbreviations

The following abbreviations are used in this manuscript:

HZ	Herpes zoster
VZV	Varicella-zoster virus
RT-PCR	Real-time polymerase chain reaction
CSF	Cerebrospinal fluid
NIID	“Prof. Dr. Matei Balș” National Institute of Infectious Diseases
LOS	Length of stay
IQR	Interquartile range
OR	Odds ratio
CI	Confidence interval
